# A Critical Review of Analytical Methods for the Quantification of Phthalates Esters in Two Important European Food Products: Olive Oil and Wine

**DOI:** 10.3390/molecules28227628

**Published:** 2023-11-16

**Authors:** Flávia Freitas, Maria João Cabrita, Marco Gomes da Silva

**Affiliations:** 1 LAQV/REQUIMTE, Department of Chemistry, NOVA School of Science and Technology, NOVA University Lisbon, 2829-516 Caparica, Portugal; fs.freitas@campus.fct.unl.pt; 2 MED—Mediterranean Institute for Agriculture, Environment and Development & CHANGE—Global Change and Sustainability Institute, Institute for Advanced Studies and Research, Universidade de Évora, Pólo da Mitra, Ap. 94, 7006-554 Évora, Portugal; 3 MED—Mediterranean Institute for Agriculture, Environment and Development & CHANGE—Global Change and Sustainability Institute, Departamento de Fitotecnia, Escola de Ciências e Tecnologia, Universidade de Évora, Pólo da Mitra, Ap. 94, 7006-554 Évora, Portugal

**Keywords:** phthalates esters, analytical methods, olive oil, wine

## Abstract

Phthalic acid esters (PAEs) are a class of chemicals widely used as plasticizers. These compounds, considered toxic, do not bond to the polymeric matrix of plastic and can, therefore, migrate into the surrounding environment, posing a risk to human health. The primary source of human exposure is food, which can become contaminated during cultivation, production, and packaging. Therefore, it is imperative to control and regulate this exposure. This review covers the analytical methods used for their determination in two economically significant products: olive oil and wine. Additionally, it provides a summary and analysis of information regarding the characteristics, toxicity, effects on human health, and current regulations pertaining to PAEs in food. Various approaches for the extraction, purification, and quantification of these analytes are highlighted. Solvent and sorbent-based extraction techniques are reviewed, as are the chromatographic separation and other methods currently applied in the analysis of PAEs in wines and olive oils. The analysis of these contaminants is challenging due to the complexities of the matrices and the widespread presence of PAEs in analytical laboratories, demanding the implementation of appropriate strategies.

## 1. Phthalates Esters in Olive Oil and Wine

Olive oil and wine are daily staples in global consumption, and when enjoyed in moderation, they form the essential cornerstones of a wholesome Mediterranean diet. This is owing to the presence of macro and micronutrients endowed with antioxidant properties, including phenols and tocopherols [1]. It is estimated that around 3 million tons of olive oil and approximately 250 million hectoliters of wine are consumed worldwide each year [2,3].

**Figure 1:**
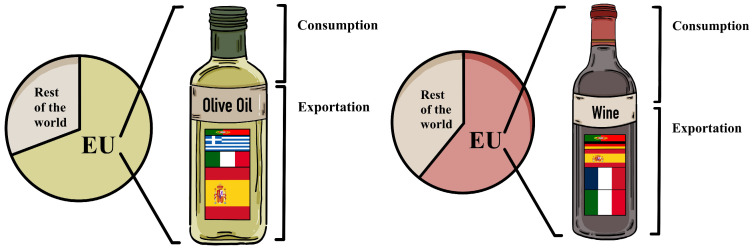
Data on the production, consumption, and export of olive oil and wine in the European Union.

The European Union (EU) is the world’s primary producer, consumer, and exporter of olive oil. The primary member states engaged in the production and export of olive oil are Spain, Italy, Greece, and Portugal. Outside the EU, this role is taken on by Morocco, Tunisia, Turkey, and Syria. The EU produces about 70% of the world’s olive oil and is responsible for 70% of global olive oil exports, with the United States, Brazil, and Japan being the main markets (Figure 1) [4].

When it comes to wine, the EU is responsible for approximately 60% of the world’s production, with Italy, France, and Spain being the countries with the highest production. Together, these three countries account for about 60% of global exports. Beyond the EU, the United States, Australia, Chile, and Argentina are the countries with the highest global production and exports (Figure 1) [5].

Therefore, due to the significant consumption, production, and interest in these two products, the importance of studying and ensuring food control becomes evident, aiming for sustainability and a better quality of life.

Olive oil is directly obtained from olive fruits only through mechanical/physical processes that do not fundamentally alter oil composition. Wine is obtained from the fermentation of fresh grapes or juice, so its composition is also affected by biochemical processes.

In the past, most of the tools and equipment used in the production of these foods were produced from conventional materials, such as wood, metal, rock, ceramics, glass, and fabric, among others. However, over time, they have been replaced by large machinery that contains various plastics, also known as polymers, in their composition. Additionally, the packaging of these raw materials has been altered. While these products were once packaged in glass containers, it is now common to find them packaged in plastic materials [6,7,8].

Over the past few decades, the use of plastic materials has played a crucial role in the daily life of society [9]. Various types of polymers are used, such as polyethylene (PE), polyvinylidene chloride, polyvinyl chloride (PVC), polyethylene terephthalate (PET), ethylene vinyl alcohol (EVOH), polypropylene (PP), and polystyrene (PS), among others [10,11].

The choice of polymer will depend on various factors, such as cost-effectiveness, recyclability, and legal requirements. Its intended purpose also plays a role in the selection process. For example, whether it is for quick heating/reheating, frozen or ambient temperature storage, cooking in a bag or not, requiring heat stability, printability, durability, or several barrier properties (e.g., water, oxygen, carbon dioxide) [12,13].

Therefore, to make the applicability of these polymers more common in various uses and as versatile as possible, additives have been incorporated to enhance their properties. Phthalate esters or di-esters are among the most widely used additives by the polymer industries, and their high demand has increased to the point that, nowadays, approximately 6 million tons are produced annually [14]. Although these plasticizers have been extensively used for over 50 years, recent years have seen increasing research into the toxicity of various environmental pollutants, including phthalates esters. Moreover, several studies have reported potential health risks associated with these substances for human health [15].

Phthalic acid esters (PAEs) are colorless and odorless substances with low solubility in water, high solubility in lipid compounds, and present low volatility. They are capable of imparting a wide range of properties to materials, such as extreme rigidity or flexibility, opacity or transparency, coloration or translucency, and the ability to withstand high or low temperatures, among other characteristics. This wide range of property variation conferred by these plasticizers allows polymers to be used in different areas and applications, especially in industrial engineering (manufacturing rigid pipes and tubes) and the food industry (packaging and films for food packaging) [16,17].

**Figure 2:**
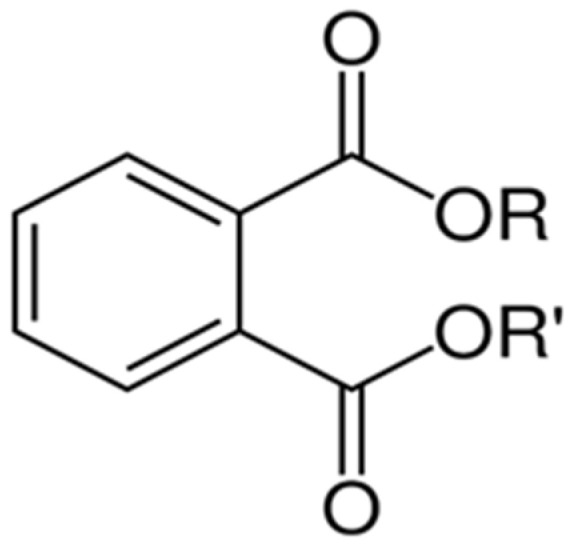
General chemical structure of phthalic acid esters. R and R′ denote linear and/or branched alkyl chains.

Their structure consists of a benzene ring linked to two ester groups in the ortho position, resulting in two aliphatic chains (Figure 2).

Depending on their substitution, this can generate more than 60 different types of PAEs with distinct properties (Table 1) [16].

It is already known that the main sources of human exposure to PAEs are oral (through food, pacifiers, baby bottles), inhalation (air contaminated by building materials, accidental inhalation of soil, household dust, PVC-based medical devices), and dermal contact (creams, shampoo, soaps) with products containing these substances [17,18].

However, the most significant source of exposure is food intake, as it can absorb compounds that migrate from plastic packaging to the food matrix or become contaminated during the production process [19,20].

This phenomenon is due to the fact that phthalates do not chemically bind to the polymer matrix, leading to their easy migration over time through exposure, increased temperature, and mechanical stress, among other factors [12,17].

As a result, these compounds can migrate into food through the typically used manufacturing processes, packaging films, gloves used for food preparation, and storage containers. These compounds have also been found in the inks and adhesives of food packaging, as well as in coatings for kitchen utensils [19,21].

The risk of migration is more pronounced in nonpolar foods, such as olive oil, due to the lipophilic nature of phthalates. This is because phthalates have a specific affinity for fatty and nonpolar substances, which makes their migration easier.

### 1.1. Toxicity

**Figure 3:**
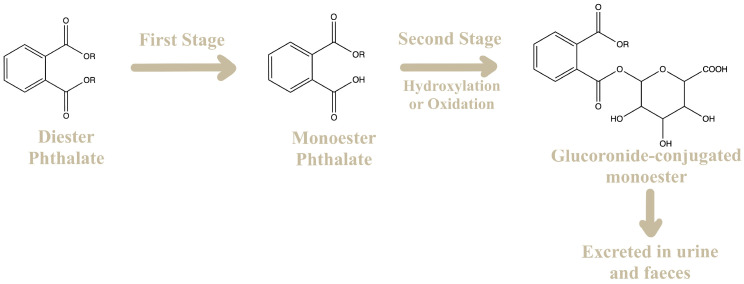
Metabolic pathway of phthalic acid esters.

Due to the lipophilic nature of PAEs, adsorption can occur through dermal and pulmonary tissues. However, the primary route of absorption occurs in the saliva or stomach after oral administration. In mammals, the metabolism of PAEs is rapid, and their distribution occurs uniformly throughout the body [22,23,24]. Phthalates esters undergo a biotransformation pathway that occurs in two stages, Figure 3.

In the first stage, lower molecular weight phthalates are hydrolyzed to form monoester phthalates through biotransformation catalyzed by lipases and esterases in the intestine and parenchyma. Typically, this initial metabolic step is associated with detoxification. However, in vitro and in vivo studies have shown that diester phthalates become more biologically active when they undergo hydrolysis and convert into monoester phthalates. On the other hand, higher molecular weight phthalates can be metabolized to form oxidative products. In the second stage, known as conjugation, both the hydrolyzed and oxidized monoesters can react with glucuronic acid catalyzed by the enzyme uridine 5′-diphospho-glucuronyl transferase. Glucuronidation facilitates excretion and can reduce the bioavailability of metabolites, minimizing their potential biological activity [25,26].

Relatively polar and short-chain phthalates (up to eight carbons), such as DMP and DEP, are rapidly hydrolyzed and have an elimination half-life in their free glucuronidated form of about 5–6 h. However, long-chain phthalates, like DEHP and DiNP, have a longer elimination half-life. Only 2–7% of DEHP is semi-eliminated from the human body in approximately 12 h [25,27].

### 1.2. Health Risks

**Figure 4:**
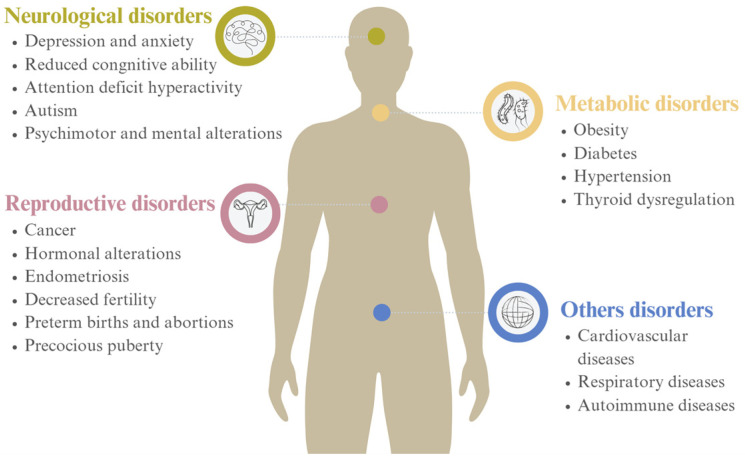
Negative health effects caused by PAEs in human health.

Due to their unique physicochemical properties, certain phthalates and their metabolites have a severe toxic effect on human health, especially on the reproductive, endocrine, and respiratory systems (Figure 4). Several studies report that the accumulation of phthalates in the body disrupts growth and reproduction, as well as induces genotoxicity, neurotoxicity, and carcinogenicity [20,28,29,30,31].

In general, phthalates demonstrate low acute toxicity in animals, with median lethal dose (LD_50_) values ranging from 1 to 30 g/kg of body weight or above. In subchronic studies with rodents, phthalates induced dose-related adverse effects in the liver, kidneys, thyroid, and testicular tissue [17].

These studies have led several countries to intervene and regulate exposure to phthalates and other substances.

### 1.3. Regulation

Due to the increasing focus on consumer food safety in Europe, strict requirements for the use of food contact materials (FCMs) have been implemented, as outlined in Regulation (EC) No 1935/2004 [32]. This regulation emphasizes that any material or product intended to come into direct or indirect contact with food must not transfer chemical substances to food products in amounts that could pose a risk to human health or result in unacceptable changes in the composition of these foods or the deterioration of their organoleptic properties.

Furthermore, specific migration limits (SMLs) have been established for five permitted phthalates (DEHP, BBP, DBP, DINP, and DIDP) in FCMs, based on a toxicological assessment outlined in Annex I of Regulation (EU) 10/2011 [33]. In 2019, the European Food Safety Authority (EFSA) also defined a tolerable daily intake (TDI) of 50 µg/kg of body weight per day for DBP, BBP, DEHP, and DINP, and 150 µg/kg for DIDP [34].

It is worth noting that, although DIBP is not authorized as an additive for FCMs, it may be present in these materials in small quantities as an impurity or because of its use as an adjuvant in the manufacturing process of certain types of plastics [35].

In 2023, the EU reviewed these data and established new SMLs, implementing Regulation (EU) 2023/1442 [35], which amends Annex I of Regulation (EU) 10/2011 (Table 2).

This amendment came into effect on 1 August 2023. However, plastics in contact with food that were in compliance with the FCM regulation before the amendment’s entry into force and were placed on the market before 1 August 2023 can remain on the market until their stocks are exhausted [35].

Despite this, there is still no specific regulation for the permitted quantity of phthalates in food. Therefore, even though SMLs are controlled in packaging and other materials, it is necessary to identify the sources of migration of these plasticizers into food. If they are found to be contaminated, it indicates that they have come into contact with one or more materials that are not suitable during their production process.

In the case of wine and olive oil, several studies report that contamination with phthalates can occur both during the production and treatment of the fruit, as they are often used in harvesting nets, pipes, tanks, and other plastic materials, as well as during storage using synthetic corks and plastic containers [36,37].

Even the drinking water used for irrigation or for washing production materials can be contaminated with these plasticizers [38]. For example, the World Health Organization (WHO) recommends a maximum concentration of 8 µg/L for DEHP [39]. This compound, in particular, is a global issue and has become an omnipresent pollutant in the environment, particularly in food. This is the most commonly detected and/or quantified phthalate as it is used in the production of flexible plastics like PVC and PET, which are commonly used for producing caps and single-dose sachets [40,41].

In 2011, Taiwan reported the “largest episode of food contamination with plasticizers in human history,” and various contaminated foods were found in the market [42]. This incident, along with new studies on the hazards of phthalates to human health, has made this food safety issue a global concern. Therefore, it has become imperative to reconsider internationally accepted regulations to mitigate this problem in food products.

There are various regulations for phthalates in different parts of the world, and as a result, it is expected that there will be substantial variation in phthalate concentrations in foods depending on the region where they are produced. This makes phthalate contamination an increasingly cross-border food safety issue as the global market expands.

Due to the widespread use of plastics, it is impractical to completely eliminate the source of contamination. Since it is not possible to remove these products from the global market, more and more research is focusing on the removal of these plasticizers. Wang et al. reviewed the methods for removing PAEs from food [38]. For polar food matrices like drinking water and beverages, methods such as physical and chemical adsorption, microbial degradation, membrane filtration, and chemical oxidation, among others, are typically used. However, for non-polar food matrices, like vegetable oils, methods such as physical adsorption, steam distillation, molecular distillation, and solvent extraction are employed. Nevertheless, for the latter, research in this area is limited, primarily due to the significant susceptibility of vegetable oil quality to external conditions. It is important to note that regardless of the matrix, PEs can only be removed to a certain extent [38]. 

Finally, one believes it is more advantageous to review the materials used during the harvesting, production, and packaging of food matrices, incorporating strategies to prevent contamination by plasticizers.

Certainly, the quantity of phthalates entering the human body solely through the consumption of olive oil or wine is exceedingly minimal and might not reach levels capable of inducing toxicological effects. Nevertheless, when considering the cumulative exposure to all plasticizers found in various elements of dietary intake, the potential risks to human health should not be casually dismissed.

## 2. Identification and Quantification of Phthalate Esters in Olive Oil and Wine

It is well known that human exposure to foods containing PAEs is daily, leading to accumulation in the body and resulting in long-term harmful effects.

The development of analytical methods that allow the identification and quantification of these compounds at low concentration levels, especially in food matrices consumed in large quantities, is urgently needed.

Traditionally, the analysis of phthalates is commonly performed using either gas chromatography (GC) or liquid chromatography (LC), often followed by mass spectrometry (MS) detection. Other analytical techniques have also been used, such as ultraviolet spectrophotometry (UV), Raman spectroscopy, flow-injection chemiluminescence (FI-CL), and more recent methods employing enzyme-linked immunosorbent assay (ELISA) and Polymerase chain reaction (PCR), as shown in Table 3.

However, due to the complex nature of real matrices, direct injection into analytical systems is not advantageous, and therefore, prior sample preparation is required. This preparation depends on the physicochemical characteristics of the matrix, the target compounds, and the aimed concentration levels.

In addition to the complexity of the matrices in which they are present, phthalates are generally found at low concentrations, typically in the range of µg/L (ppb level) or less, often falling below the limit of detection (LOD) of instruments. It is almost strictly necessary to employ both extraction and cleaning/pre-concentration steps to maximize analyte recovery and minimize the presence of potential interferents [43,44,45].

As described in Table 3, different analytical pre-treatment approaches for the analysis of phthalates in olive oils and wines have been reported in the literature. These include liquid–liquid extraction (LLE), solid-phase microextraction (SPME), solid-phase extraction (SPE), molecularly imprinted solid-phase extraction (MISPE), magnetic solid-phase extraction (MSPE), dispersive liquid–liquid microextraction (DLLME), and quick, easy, cheap, effective, rugged, and safe (QuEChERS) methods, among others.

Due to their simplicity, customization, and automation capabilities, classical techniques, like LLE, with or without additional clean-up steps, are still preferred when it comes to phthalate esters extraction to isolate and concentrate target analytes. However, these methods have several limitations. They typically demand a substantial amount of time, intensive labor, and the use of significant volumes of potentially harmful.

Research and the advancement of alternative techniques, such as Solid-Phase Microextraction (SPME) and Liquid-Phase Microextraction (LPME), have demonstrated the capacity to mitigate certain limitations, all the while preserving elevated extraction efficiency and analyte enrichment levels.

Thus, given the worldwide importance of olive oil and wine in society and the growing interest in the analysis of food contaminants, a comprehensive effort has been made to address all articles published on the analysis of phthalate esters in olive oil and wine, excluding other vegetable oils and alcoholic beverages.

**Table 3:** Presents the analytical techniques reported in the literature for phthalate detection in the last 20 years, organized by matrix (wine and olive oil), phthalates esters analyzed, sample preparation, and analytical technique used. Limits of detection (LOD), quantification (LOQ), and Recoveries obtained in each study are represented, as well as the concentration of phthalates esters found in real samples. Studies on wine are represented in dark-shaded areas, and in light-shaded areas are the studies on olive oil.

PAEs	Sample Preparation	Analytical Technique Column	LOD	LOQ	Recovery %	Concentration of PAEs	R *
DMP, DEP, DIBP, DBP, BBP, DCHP, DEHP, DOP, DINP, DIDP	LLE Isohexane	GC/MS Agilent DB5- MS (30 m × 0.25 mm × 0.25 μm)	0.004–0.020 mg/L	0.01–0.05 mg/L	98–100	0.008–0.273 mg/kg DBP, BBP, DEHP	[46]
DEP	LLE 1,1,2-trichlorotrifluoroethane	GC/MS Varian VF-Xms column (29.3 m × 0.25 mm × 0.25 μm)	0.7 mg/L	2.6 mg/L	103.9–110.4	<LOD	[47]
DMP, DEP, DIPrP, DAP, DPrP, DIBP, DBP, DMEP, DIPP, BMPP, DEEP, DPP, DHXP, BBP, DBEP, DCHP, DEHP, DHP, DPhP, DNOP, DINP, DIDP, DNP	LLE Acetonitrile	LC/MS/MS Agilent Poroshell 120 EC-C18 (100 × 4.6 mm × 2.7 µm)	0.8–15 µg/kg	10–100 µg/kg	75.5–113.3	ns	[48]
DBP	LLE Hexane	FI–CL	0.03 pg/mL		96–103.3	0.09–0.22 µg/mL	[49]
DBP	LLE Hexane	icELISA	64.5 ng/mL		83.1–101.7	ns	[50]
DEP	LLE Hexane	GNP-rt-IPCR	1.06 pg/L		96.65–110.02	41.88 µg/kg	[51]
DMP	LLE Hexane	BA-rt-IPCR	1.98 pg/L		88.18–108.99	86.96–182.85 µg/L	[52]
DMP, DEP, DIBP, DBP, BBP, DEHP, DOP, DINP, DIDP	Filter 0.2 µm	HPLC/MS/MS Phenomenex 75 mm Synergi Hydro-RP (2 mm × 4 µm × 4 mm)	0.5–8.8 µg/L	1.6–26.6 µg/L	94.6–105.7	1.8–10.7 µg/L DIBP, DBP, BBP	[53]
DMP, DEP, DBP, BMEP, DPP	MA-LLME	GC/MS Teknokroma TRB-624 (30 m × 0.25 mm × 1.40 μm)	0.1–0.4 µg/L	0.3–1 µg/L		4.2–25 µg/L DBP, DPP, DEP	[54]
DMP, DEP, DBP, BBP, BBP, DEHP	USVADLLME	GC/MS Lab-made SE-54 (30 m × 250 μm × 0.25 μm)	0.022–0.1 µg/L	0.075–0.335 µg/L	85–100.5	11.5–312.4 pg/µL DBP, BBP, DEHP	[55]
DBP, BBP, DEP, DIOP	DLLME	GC-FID Lab-made SE-54 (15 m × 0.25 mm × 0.33 μm)	0.34–0.78 µg/L		70–120	1.2–5.8 µg/L BBP, DBP	[56]
DBP, BBP, DCHP, DEHP, DOP	UA-DLLME-SFOD	GC-FID Agilent HP-5 (30 m × 0.250 mm × 0.25 μm)	0.64–2.82 µg/L	1.93–8.47 µg/L	75–98	ns	[57]
DIBP, DBP, BBP, DEHP	IL-DLLME [C8MIM] [PF6]	HPLC/DAD Waters Xterra C18 (15 cm × 4.6 mm × 5 μm)	1.5–2.2 ng/mL	5–7.3 ng/mL	91.6–10.6	0.018–0.122 µg/mL DIBP DBP	[58]
DMP, DEP, DBP, DEHP, BBP, DOP	HS-SPME PDMS	GC/MS Varian CP-WAX 52 CB (30 m × 0.32 mm × 0.25 µm)	16–35 ng/L		72–121	0.3–7.40 µg/L DMP, DEP, DBP, DEHP, BBP, DOP	[36]
DMP, DEP, DBP, DEHP, BBP, DOP	HS-SPME CW-DVB; PDMS-DVB with sodium chloride	GC/MS Varian CP-WAX 52 CB (30 m × 0.32 mm × 0.25 µm)	0.06–2.9 µg/L	0.1–4.2 µg/L	64–135	Total ranging from 7–12 ng/mL	[59]
DBP, BBP, BDE, DOP	HS-SPME PDMS-DVB with sodium chloride	GC/MS SGE HP-5 (60 m × 0.25 mm × 0.25 μm)	0.03–0.11 µg/L	0.09–0.36 µg/L	80.3–107.6	0.71–20.8 µg/L DBP, DOP	[60]
DEP, DBP, DEHP	DI-HF-SPME MWCNTs/SiO2 reinforced hollow fibre	GC/MS Thermo TR-5 MS (30 m × 0.25 mm × 0.25 μm)	0.006–0.03 ng/mL	0.02–0.1 ng/mL		<LOD	[61]
DBP	DI-SPME Graphene oxide	GC/MS HP-5 MS (30 m × 0.25 mm × 0.25 μm)	0.3 ng/L		98	<LOD	[62]
DMP, DEP, DBP, DIBP, BBP, DEHP	SPE Amberlite XAD-2	GC-FID Lab-made SE-54 (15 m × 0.25 mm × 0.24 μm)	1.21–2.51 pg/µL	2.42–5.03 pg/µL	94–103	4.9–12.3 pg/µLDBP, DEHP	[63]
DMP, DEP, DBP, BCEP, BBP, DEHP	SPE Carbograph 1	GC/MS Lab-made SE-54 (30 m × 250 μm × 0.23 μm)	0.2–14 ng/mL	0.5–25 ng/mL	78–105	0.1–23 ng/mL DBP, BBP, DEHP	[64]
DMP, DEP, DEHP, DIBP, DBP, BBP	SPE C18	GC/MS Restek RTX-5MS (30 m × 0.25 mm × 0.25 µm)	0.015–0.018 µg/mL	0.024–0.029 µg/mL	33–109	0.025–0.276 µg/mL DIBP, DBP, BBP, DEHP	[65]
DBP, DEHP, DEP	Filter; SPE 0.45 µm; C18	LC/DAD Poroshell 120 EC C18 (4.6 mm × 50 mm × 2.7 µm)	0.25–0.38 ng/mL	0.75–1.10 ng/mL		23.6–334 ng/mL DBP, DEHP, DEP	[66]
BBP, DEP, DBP, DMP	MIP-SPE	HPLC/MS Agilent ZORBAX Eclipse XDB-C8 (50 mm × 2.1 mm × 3.5 µm)	0.03–0.20 µg/L	0.09–0.68 µg/L	74–98	0.3–5 µg/L BBP, DEP, DBP, DMP	[67]
DBP, BBP, DEHP	QuEChERS	GC/MS J&W DB-5MS (30 m × 0.25 mm × 0.25 μm)	0.08–2.25 ng/mL		104–123	1.69–9.72 ng/mL DBP, BBP	[68]
DMP, DEP, DIBP, DBP, DHP, BBP, DCHP, DEHP, DNOP, DINP, DIDP	LLE Acetonitrile	GC/MS/MS Restek Rxi-5Sil MS (30 m × 0.25 mm × 0.25 μm)	0.004–0.130 mg/L	0.012–2.600 mg/L	90.1–108.2	0.03–7.52mg/kg DIBP, DBP, BBP, DEHP, DINP	[69]
DMP, DEP, DIBP, DBP, BBP, DHP, DEHP, DOP, DINP, DIDP	LLE Acetonitrile	GC/MS Supelco SPB-5MS (30 m × 0.25 mm × 0.25 mm)	0.003–1.2 mg/kg	0.010–4.0 mg/kg	93.5–99.4	0.060–6.249mg/kg DMP, DEP, DIBP, DBP, BBP, DEHP, DINP, DIDP	[70]
DMP, DEP, DBP, DEHP	LLE by means of the carbon nanotube Pseudophase Distilled water; MWCNTs	GC/MS Supelco SLB-5 ms (30 m × 0.25 mm × 0.25 μm)	25–50 μg/L		92–104	0.15–5.1 mg/L DMP, DEP, DBP, DEHP	[71]
DMP, DEP, DPP, DIBP, DBP, BBP, BMPP, DEHP, DOP	LLE Acetonitrile	GC/MS/MS Restek Rxi-5ms (30 m × 0.25 mm × 0.25 µm)	0.43–1.67 μg/L	1.48–5.75 μg/L	89–114	0.17 mg/kg DEHP	[72]
DMP, DEP, DIBP, DBP, DMEP, BMPP, DEEP, DPP, DHXP, BBP, DBEP, DCHP, DEHP, DPhP, DOP	LLE hexane saturated acetonitrile and hexane	GC/MS/MS Agilent HP-5MS (30 m × 0.25 mm × 0.25 μm)	0.1–4.0 μg/kg		70.0–110.8	ns	[73]
DEHP, DBP, DIBP, DINP	Dilution Hexane	GC/MS Lab-made pre-column OV-1701-OH (0.5 m × 0.25 mm × 0.05 mm) in series w/2 lab-made columns OV-61-OH (2.5 m × 0.32 mm × 0.20 µm) OV-225-OH (15–20 m × 0.25 mm × 0.20 µm)	10 μg/kg - 1 mg/kg	40 μg/kg - 3 mg/kg	82–106	90–6480 μg/kg DEHP, DINP, DBP	[74]
DMP, DEP, DPP, DBP, BBP, DCHP, DEHP, DINP, DIDP	Dilution Hexane	GC×GC/MS/MS 1D Merck SLB-5 ms (10 m × 0.25 mm × 0.10 μm) 2D Merck SLB-35 ms (1.5 m × 0.10 mm × 0.10 μm)	0.02–0.63 mg/kg	0.06–2.10 mg/kg		0.22–8.0 mg/kgDPP, DEHP, DINP, DIDP	[75]
DMP, DEP, DPP, DBP, BBP, DCHP, DEHP, DINP, DIDP,	Dilution Hexane	GC/MS/MS Equity-5 (5 m × 0.53 mm × 0.53 μm)	0.004–0.341 mg/kg	0.013–1.136 mg/kg		0.018–55.9 mg/kgDEHP, DIDP DBP, DPP, DINP, DEP	[76]
DMP, DEP, DIPrP, DAP, DPrP, DIBP, DBP, DMEP, DIPP, BMPP, DEEP, DPP, DHXP, BBP, DBEP DCHP, DEHP, DHP, DPhP, DNOP, DINP, DIDP, DNP,	LLE Acetonitrile	LC/MS/MS Agilent Poroshell 120 EC-C18 (100 × 4.6 mm × 2.7 µm)	0.8–15 μg/kg	10–100 μg/kg	82.2–112.6	ns	[48]
BBP, DBP, DEHP, DEP, DIBP, DIDP, DINP, DMP, DHXP, DOP, DAP, DPP	LLE Hexane saturated acetonitrile	UHPLC/MS Thermo Accucore aQ C18 (2.6 μm × 2.1 × 100 mm)	0.02–0.35 mg/kg	0.07–1.17 mg/kg	79–109	0.3–256.2 mg/kg DEHP, DIDP, DINP, DMP, DNOP, BBP, DEP, DIBP	[77]
DMP, DEP, DAP, DPrP, DIBP, BBP, DBP, DCHP, DHXP, DEHP	LLE Acetonitrile	UHPLC/MS/MS Thermo Syncronis C18 (100 × 2.1 mm, 1.7 µm)	0.1–1 μg/kg	0.3–3.3 μg/kg	85.1–95.5	3.0–309 μg/kg DMP, DEP, DIBP, BBP, DBP, DEHP	[78]
PAEs hydrolyzed in Phthalic Acid	LPME Tributyl phosphate	HPLC/MS/MS GL Sciences Inertsil ODS-3 (250 mm × 4.6 mm × 5 m)	1 μmol/kg	1.3 μmol/kg	86–107	4.82 μmol/kg	[79]
DMP, DEP, DPP, DIBP, DBP, BBP, DCHP, DEHP, DINP, DIDP,	LLE and DI-SPME Acetonitrile; PDMS	GC/MS/MS Supelco SLB-5ms (10 m × 0.1 mm × 0.1 mm)		0.015–0.144 mg/kg		0.228–7.207 mg/kg DEP, DIBP, DBP, DEHP, DINP, DIDP,	[80]
DMP, DEP, DAP, DIBP, DBP, BBP, DCHP, DEHP	LLE and SPME Acetonitrile; MIL-88(Fe)/Go	GC-FID Agilent HP-5 (30 m × 0.32 mm × 0.25 μm)	0.5–2 ng/g	1.7–6.7 ng/g	83.1–104.1	<LOD	[81]
DPP, DBP, DEHP	HS-SPME G/PVC nanocomposite	GC-FID Varian CP-Sil 8 CB (30 m × 0.32 mm × 0.25 µm)	0.06–0.08 μg/L	0.2–0.3 μg/L	87–112	<LOD	[82]
DMP, DEP, DIBP, DBP, DMEP, 1,2MPP, 1,3MPP, DEEP, DAP, DHP, BBP, BBEP, DCHP, DEHP, DOP, DNP	SPME DVB/CAR/PDMS	GC/MS/MS Phenomenex Zebron ZB-5ms (30 m × 0.25 mm × 0.25 m)	0.02–0.05 mg/kg			87–840 μg/kg DIBP, DBP, BBP, DEHP	[83]
DMP, DEP, DBP, BBP, DEHP	LLE and SPE Acetonitrile; PSA	GC/MS Thermo TG- 5MS column (30 m × 0.25 mm × 0.25 µm)	0.10–0.79 μg/kg	0.33–2.6 μg/kg	72,4–103	0.05–1.28 mg/kg DMP, DBP, BBP, DEHP	[84]
DBP, BBP, DEHP	LLE and SPE Acetonitrile; Florisil	GC/TOFMS Agilent DB-5MS column (30 m × 0.25 mm × 0.25 μm)	4.70–10 μg/kg	14.2–30.4 μg/kg	83.9–97.8	13.2–729 μg/kgDBP, DEHP	[85]
DMP, DEP, DBP, DIBP, DEHP, BBP, DINP, DIDP	LLE and SPE Acetonitrile and tetrahydrofuran; Alumina	GC/MS HP-5MS (30 m × 0.25 mm × 0.25 μm)	2–170 ng/mL	6–500 ng/g	62–110	<LOD	[86]
DMP, DEP, DIPrP, DPrP, DIBP, DBP, DMEP, DIPP, BMPP, DEEP, DPP, DHXP, BBP, DBEP, DCHP, DHP, DPhP, DEHP, DNOP, DNP	MAE-GPC-SPE C18	GC/MS/MS Agilent HP-5MS (30 m × 0.32 mm × 0.25 μm)	0.218–1.367 μg/kg	0.72–4.51 μg/kg	93.04–104.7	0.42–0.70 mg/kg DBP, DEHP	[87]
DMP, DEP, DPP, DBP, BBP, DOP	LLE and SPE Hexane; Florisil	GC/MS Santa Clara HP-5MS (30 m × 0.25 mm × 0.25 μm)	0.002–0.004 mg/L	0.006–0.012 mg/L	87–102	0.049–2.295 mg/L DMP, DEP, DBP, DPP, BBP, DOP	[88]
DMP, DEP, DIBP, DBP, BMPP, DEEP, DPP, DHP, BBP, DBEP, DCHP, DHP, DPhP, DOP, DNP	LLE and SPE (QuEChERS modified) Methanol; GCB and PSA	GC/MS/MS DB-5MS (30 m × 0,25 mm × 0,25 μm)	0.02–8 μg/kg	0.07–26.68 μg/kg	70.11–115.33	0.10–1.85 mg/kg DIBP, DHP	[89]
DMP, DEP, DPrP, DAP, DIBP, DBP, DPP, DHXP, BBP, DHP, DEHP, DPhP, DNP, DDP	LLE and dSPE Acetonitrile; Q-sep QuEChERS	GC/MS Shimadzu SHRXI-5MS (30 m × 0.25 mm × 0.25 μm)	1.4–7.5 μg/kg	4.8–25.1 μg/kg	60.9–101.3	14–6166 μg/kg DMP, DEP, DAP, DPP DIBP, DBP, DPP, DHXP, BBP, DEHP, DNP, DDP	[37]
DEP, DIBP, DBP, BBP, DEHP, DOP, DINP, DIDP	LLE and SPE Acetone: methanol; DSC-18	HPLC-MS/MS Phenomenex Kinetex C18 (50 mm × 2.1 mm × 5.0 µm)		5.5–110 μg/kg	42–100	0.014–4.7 mg/kg DIBP, DBP, BBP, DEHP, DOP, DINP, DIDP	[90]
DEP, DIBP, DBP, BBP	LLE and SPE (QuEChERS) Acetonitrile; PSA	HPLC/DAD	6–9 ng/g	18–29 ng/g		<LOD	[91]
DEP, DBP, BMPP, DEEP, DNPP, DHXP, BBP, DBEP, DCHP, DEHP, DNOP, DMP, DMEP, DPP, DINP, DIDP	SPE Florisil	LC-MS/MS Agilent ZORBAX SB-C18 (10 cm × 3.5 μm × 2.1mm)	0.5–25 μg/kg	1.4–65 μg/kg	50.94–140.83	ns	[92]
BBP, DEHP	LLE and SPE (QuEChERS) Acetonitrile; PSA	SFC-UV Thermo Acclaim 120 C18 (5 μm, 4.6 mm × 250 mm	0.09–0.12 μg/mL	0.30–0.39 μg/mL	80.3–106.4	<LOD	[93]
DMP, DEP, DBP, BBP, DEHP, DOP	GPC Cyclohexane: dichloromethane	GC/MS/MS Varian Factor Four 5-ms (30 m × 0.25 mm × 0.25µm)	0.1–148 μg/kg	0.2–182 μg/kg		0.029–4.70 mg/kg DBP, BBP, DEHP	[41]
DBP, DEHP		Raman spectroscopy with SERS	At a concentration of 0.2 mg/kg, the peaks for both plasticizers were still clearly detectable			ns	[94]

* Reference: ns—not specified.

Out of these 52 studies, 90% use chromatographic analytical techniques, where 25% apply liquid chromatography and 67% apply gas chromatography. However, these techniques, besides having long analysis times and sometimes complex instrumentation, often do not provide all the information present in a sample. It may not be possible to separate and identify compounds in complex samples, especially when multiple analytes share the same retention time [95].

The identification and quantification of phthalates are also very challenging due to the issue of cross-contamination, which is a recurring problem in sample preparation, extraction/cleanup, and concentration, as well as in the chromatographic system. To address this problem, rigorous laboratory cleaning and handling procedures are typically applied, and internal standards, often isotopically labeled, are used to reduce matrix effects and correct potential variations during the analyses [44,45].

### 2.1. Sample Preparation

In recent years, there has been an increasing demand for new extraction techniques that can be automated and reduce both extraction times and the use of organic solvents. This aims to prevent environmental contamination in analytical laboratories and, most importantly, reduce the costs associated with sample preparation, contributing to greener analytical chemistry [96,97].

#### 2.1.1. Liquid–Liquid Extraction

Liquid–liquid extraction (LLE), also known as solvent extraction, is one of the oldest and simplest extraction techniques and one of the most commonly used for the analysis of phthalate esters in food matrices. This technique is based on the separation of target analytes with different solubilities in two immiscible solvents. It is commonly used in aqueous samples to pre-concentrate and remove unwanted compounds from the matrix [98,99].

The choice of solvent, the volume used, and the affinity of the target compounds for the extraction solvent will determine the efficiency and duration of the technique. Generally, extraction efficiency increases with the use of larger volumes of extraction solvent; however, this will reduce the concentration of target analytes in the solution. To mitigate this problem, multiple extractions with smaller volumes are often performed [98,99].

However, the use of this simple method has significant disadvantages, such as its unsuitability for hydrophilic compounds, the formation of emulsions that hinder complete recovery of the extract, the recurring use of large amounts of organic solvents leading to significant hazardous waste disposal, and the difficulty of automating the entire process. Another drawback of this technique is that its selectivity is not as specific as some other methods, as it tends to extract undesired analytes from the matrix under study. But, this disadvantage can be an advantage for non-targeted analyses [98,99].

LLE is the most widely used method for the extraction of phthalates in both wines and olive oils. Several studies report LLE of phthalates in wine and olive oils using solvents such as acetonitrile, hexane, acetone, and methanol as extraction solvents (Table 3).

For a reliable and efficient method, several parameters should be optimized during implementation. Leitz et al. optimized an LLE method for the analysis of phthalates in wines, where they studied the best extraction solvent to use, the ratio of extraction solvent volume/sample volume, and the number of extraction repetitions [47]. After optimization, 1,1,2-trichlorotrifluoroethane was chosen as the best solvent, achieving recovery values between 103.9–110.4%. However, due to its contribution to ozone depletion, this solvent’s production and use have been phased out under international agreements like the Montreal Protocol [100]. So today, following the principles of green chemistry, it would be necessary to use another solvent, such as hexane [47].

Dugo et al. used LLE with acetonitrile to extract phthalates from Italian olive oils, obtaining recoveries between 93.5 and 99.4%. In the study, it was observed that DEHP was present in higher concentrations in olive oil than allowed by the EU in food contact materials (1.5 mg/kg) [70].

However, as can be seen in Table 3, one considers that a simple LLE of olive oil, without clean-up steps and direct injection into the system, is a risk to the analytical instruments used. Conventional LLE should typically be used in conjunction with a clean-up step, such as SPE, using different phases like silica or Florisil. In the case of olive oils, clean-up steps are of utmost importance to remove co-extracted free fatty acids. Free fatty acids and phthalates have somewhat similar polarities, and when the extraction of phthalates is not well performed, fats can cause interference in chromatographic analysis or even system contamination.

Frankhauser-Noti sought to use a chromatographic methodology that would avoid these issues in the analytical system by separating the fatty matrix during injection with programmed temperature volatilization (PTV), forcing the compounds of interest to be transferred to the separation column while retaining the rest at the inlet [101].

Despite the good extraction efficiency of the LLE method, alternative extraction solutions based on the principles of green analytical chemistry are currently sought, including low volumes of organic solvents, simplicity, and speed.

#### 2.1.2. Dispersive Liquid-Liquid Microextraction

In recent years, there has been significant attention given to liquid-phase microextraction techniques (LPME), particularly DLLME. It was first described in 2006 by Rezaee et al. and can be considered a miniaturized modification of conventional LLE as it uses only a few microliters of extractant [102]. When compared to the classical technique, it offers advantages of simplicity, speed, cost-effectiveness, user-friendliness, reduced utilization of organic solvents, high recovery, high enrichment factor, and compatibility with chromatographic techniques like LC and GC [103].

The basic concept of Dispersive Liquid–Liquid Microextraction (DLLME) revolves around the dispersion of an extraction solvent (typically a non-water-miscible chlorinated solvent) and a disperser solvent (which can mix with both water and the extraction solvent, often acetonitrile) within an aqueous solution. This creates a more extensive interaction zone between the aqueous phase and the extraction solvent [102].

In 2013, Cinelli et al. established an ultrasound and vortex-assisted DLLME method for the extraction of six phthalates in wine [55]. Zhu et al., 2014, extracted four phthalates from wine using a simpler and faster DLLME method, making it an operationally easier and quicker analysis method than Cinelli’s [56]. LPME techniques help avoid the issue of large volumes of solvents used in classical liquid-liquid extraction but do not eliminate the use of toxic solvents, namely halogenated solvents, such as chloroform and carbon tetrachloride.

Therefore, new approaches to DLLME are regularly presented using ionic liquids as extractants. Zanjani et al. developed a new LPME method, known as solidification of organic drops (SFOD) assisted by ultrasound (UA-DLLME-SFOD). Using an extraction solvent with properties such as lower density than water, low toxicity, and a melting point close to room temperature, solidifies easily at low temperatures. In this technique, following the extraction process, the organic drop is solidified in an ice bath, collected using a spatula, melted, and directed for analysis [104]. Following this, Perez et al. applied this technique to extract five phthalate esters in food simulants and liquid samples, including wine [57].

In 2013, another modification of traditional DLLME using ionic liquids (ILs) was addressed for the extraction of four phthalates in wine, known as ionic liquid dispersive liquid–liquid microextraction (IL-DLLME) [58]. Ionic liquids represent a new group of organic salts that maintain their liquid state at temperatures under 100 °C and possess unique physicochemical properties, such as minimal vapor pressures, strong thermal stability, and excellent solubility for both organic and inorganic substances. In addition to being non-toxic and non-volatile, ILs are also recyclable, making them considered green extraction solvents [105,106].

Xie et al. also applied the IL-DLLME technique for the extraction of four phthalates in edible oils [91]. However, for this matrix, a clean-up step before extraction was necessary. Despite several successful applications in aqueous matrices (water, urine, blood, etc.), DLLME lacks selectivity and encounters serious co-extractant interferences in oily matrices such as olive oil. Thus, Xie applied another technique called QuEChERS [91].

#### 2.1.3. Solid-Phase Microextraction Extraction

Solid-Phase Microextraction (SPME) is an analytical technique that was invented and developed in the 1990s by Pawliszyn and associates to simplify the sample preparation procedure [107].

SPME is a rapid, simple, and effective approach for the adsorption/absorption and desorption of analytes, combining sampling, isolation, and enrichment in a single step without the need for solvents. It employs a needle, typically comprising fused silica, which is externally coated with a liquid polymer or solid sorbent material to extract analytes from a wide range of liquid or solid samples [107,108].

In the SPME technique, the property of the coating material is the most important key to enhancing its extraction efficiency since it relies on establishing the extraction equilibrium of analytes between the fiber coating and the sample based on the polarity of the target analytes. Depending on the fiber, there are two different processes for collecting volatile and non-volatile compounds: direct immersion of the fiber into the liquid sample (DI-SPME) or exposing the fiber to the headspace above the sample (HS-SPME) until equilibrium is reached [109,110].

After the required extraction time, the coated fiber containing the analytes of interest is introduced into a chromatographic system, and the analytes are desorbed. Nowadays, this technique can be automated with an autosampler in a chromatographic system, making the process of extracting, pre-concentrating, and transferring analytes to the chromatographic system an attractive and desirable method [111].

In addition to these advantages, since solvents are typically not used, SPME is considered a green technique with the significant benefit of no secondary contamination occurring during the sample pre-treatment step. Furthermore, a single fiber can be reused hundreds of times [112].

However, it has some limitations, such as the fragility of the fiber and the potential for analyte carryover during analysis if not fully desorbed during the previous injection [110,113].

Successful detection and quantification of phthalates in olive oil and wines using the SPME technique have already been reported. As phthalates are, in general, semi-volatile compounds, the HS-SPME method is preferred over DI-SPME to avoid interactions between the fiber and the sample matrix [114].

In the field of wines, Carrillo et al. compared different fiber coatings to select the most suitable one for phthalate analysis. The researchers investigated the impact of extraction temperature, salting-out effects, and sample volume. Their findings indicated that elevated temperatures promote better extraction results, the optimal sample volume decreases as the fiber’s polarity increases, and the quantity of salt required increases with the fiber’s polarity [59]. The authors also proposed the use of deuterated phthalates as internal standards to correct potential errors during sample preparation, avoid matrix effects, and improve the reproducibility of the SPME extraction methodology [36].

In the realm of olive oils, Holadová et al. evaluated four different fiber types: polydimethylsiloxane (PDMS), polyacrylate (PA), carboxen/polydimethylsiloxane (CX/PDMS), polydimethylsiloxane/divinylbenzene (PDMS/DVB), and tested various solvents as matrix modification agents to facilitate the transfer of some phthalates to the headspace. They also found that temperature and sample agitation are critical points during SPME extraction [115]. Barp et al. utilized the same SPME technique to identify and quantify phthalates in vegetable oils, studying only two different fibers and comparing direct immersion extraction with headspace extraction [80].

Rios et al. applied HS-SPME at high temperatures (250 °C) to analyze phthalates in olive oil [83]. The need to use high temperatures during sample incubation is due to the fact that some compounds, such as DNOP and DNP, have low volatility and do not easily transfer to the headspace like other compounds. However, the use of high temperatures presents challenges related to the durability of the fiber, as degradation can occur. Furthermore, the absorption/adsorption process from SPME is an exothermic process. Hence, high extraction temperatures tend to reduce the extraction efficiency. Moreover, constant monitoring and replacement of fibers when necessary are essential to maintain accurate results [83].

The main benefit of this extraction method is the absence of sample manipulation, thus avoiding potential contaminations from glassware, the environment, solvents, and samples. It is also a fast and cost-effective method compared to conventional cleaning processes, such as LLE and SPE.

#### 2.1.4. Solid-Phase Extraction

Solid-phase extraction (SPE) was first introduced in the 1970s, and due to its effectiveness and versatility, it has become one of the most widely used extraction techniques for isolating, enriching, or cleaning analytes from various matrices [116,117].

In SPE, one or more analytes from a liquid sample are separated by extraction, partitioning, and/or adsorption onto a solid stationary phase. The wide variety of sorbents with different compositions and functional groups available allows for the separation of target analytes from the original matrix, as they have a greater affinity for the sorbent material than for the solvent used. After being retained on the sorbent material, analytes can be eluted and pre-concentrated using an appropriate solvent [118].

This technique allows for concentration factors of up to 500 times, which can be extremely useful for the targeted analysis of low-concentration compounds in real matrices, such as phthalates. As shown in Table 3, several methods using SPE for phthalate extraction in olive oil and wine have been developed. Currently, the most significant interest in scientific research has been in the development of new solid sorbents to achieve higher sensitivity and reliability [118].

In the extraction of six phthalates in wines, Russo et al. used the Carbograph 1 sorbent, which allowed for recoveries between 78% and 105% [64]. Later, Cinelli et al. from the same group used the Amberlite XAD-2 resin for the first time to extract the same phthalates from beverages with a wide alcohol range (10–40%) [63]. This group investigated both breakthrough curves to study the relationship and interactions between the phthalates, eluents, and adsorbents used, as well as the presence of NaCl to improve analyte recovery. XAD2 proved to be more efficient, enabling better recoveries (94–103%) and lower limits of quantification (LOQs) [63,64]

In the field of olive oils, SPE is commonly used after LLE as a clean-up step, using different phases such as PSA, C18, or Florisil (Table 3). However, it is considered a risk to use SPE cartridges because most of them are maunfactured from polyethylene or polypropylene, which can result in the release of phthalates into the adsorbent and potential cross-contamination of the real sample. It is advisable to use glass cartridges or extraction disks [119].

In addition to conventional SPE, other adaptations have been studied, such as the application of molecularly imprinted polymers (MIPs) as SPE sorbents [120]. MIPs are tailor-made polymeric materials designed for a specific analyte. Growing in popularity in the last decade due to advancements in their synthesis that allow for increased molecular recognition, MISPE has already been applied to the extraction of four phthalates in wine. Barciela-Alonso et al. prepared the MIP via precipitation polymerization using DBP phthalate as the template, and the SPE procedure coupled with HPLC/MS proved to be a precise and sensitive method, with recovery factors ranging from 74% to 98% in wines [67]. It was not found in any study describing the use of MIPSE in olive oil, probably because the triglycerides that comprise olive oil are too chemically similar to phthalate esters in order to allow target successful extraction.

Dispersive SPE (d-SPE) is commonly used for clean-up during phthalate extraction. This technique involves dispersive mixing sorbents so that they retain the target analytes present in the analytical solutions. Subsequently, after centrifugation and removal of the supernatant, the analytes are eluted with appropriate solvents. This method was applied as a clean-up step by Bi et al. after LLE extraction to analyze the presence of 15 phthalates in vegetable oil samples, where recoveries ranged from 60.9% to 101.3% for olive oils [37].

#### 2.1.5. QuEChERS

To overcome some of the disadvantages of the traditional LLE method, either coupled or not with clean-up steps, the QuEChERS method emerged. QuEChERS, which stands for Quick, Easy, Cheap, Effective, Robust, and Safe, was first introduced by Anastassiades et al. in 2003. His group used QuEChERS to determine pesticides in fruits and vegetables [121].

This method, which is arguably the most successful development in the analysis of food contaminants in recent years, is a multi-step analytical procedure based on LLE with salting-out and d-SPE. There are five steps involved in the QuEChERS protocol. The procedure begins with the homogenization of the aqueous sample, followed by extraction with acetonitrile. Dehydration with MgSO4 or NaCl is performed to promote the separation of water from the organic solvent (salting-out effect), and then impurities are removed with a variety of sorbents (e.g., primary secondary amine, graphite carbon black, C18). After clean-up, the sample is analyzed using chromatographic techniques [121].

The rapid adoption of this simple and efficient method led to its adaptation for use with other matrices and analytes, including the determination of phthalates in food matrices, such as wine. Fasano et al. applied the QuEChERS method to extract three phthalate esters from wines packaged in laminated plastic-coated cardboard boxes (Tetra Pak). The most contaminated wine contained 9.72 µg/L of DBP [68].

In the case of olive oils, there were three articles mentioning the use of the QuEChERS method [89,91,93]. However, it appears that these authors simply followed a procedure involving LLE or UAE followed by d-SPE. They did not perform one of the main steps of the QuEChERS method: the salting-out extraction step, which promotes an equilibrium between the aqueous phase and the organic phase. This indiscriminate use of the term “QuEChERS,” where LLE would be more appropriate, is problematic and unnecessary since it can lead to confusion between the two techniques. Nevertheless, several authors use the QuEChERS method for the determination of contaminants such as pesticides, followed by chromatographic analysis in olive oils [122,123,124].

#### 2.1.6. Other Extraction/Clean-Up Methods

Although the most commonly used extraction procedures to extract and clean phthalate residues in wine and olive oil have already been mentioned in this review, other analytical approaches are also employed by some researcher teams.

For example, gel permeation chromatography (GPC), first used in the 1960’s, is a powerful cleaning method that separates analytes based on molecular size, eluting larger molecules first, followed by smaller ones [125]. GPC is highly recommended for its effectiveness in removing fats and oils and is applicable to a wide range of analytes, such as pesticides, polyaromatic hydrocarbons (PAHs), and phthalates, to clean extracts from complex samples, such as olive oil and wine [126]. However, this method has multiple disadvantages, such as the need for specialized equipment, which can be extremely expensive for some applications.

Some authors have reported the use of GPC as an additional cleaning step prior to analysis to remove interferences in wine and olive oil samples. For example, Cavaliere et al. used GPC as a cleaning step in a study aimed at determining the content of six phthalates in olive oil without the need for prior LLE or SPE cleaning after GPC [41].

On the other hand, Sun et al. used GPC coupled with Microwave-Assisted Extraction (MAE) and SPE to extract 20 phthalates from vegetable oil samples. The group sought an effective way to extract, clean, and concentrate analytes in the MAE–GPC–SPE method, overcoming lipid and pigment interference and increasing the sensitivity of their method [87]. However, the method involves very tedious and expensive steps, which probably prevents it from being used in routine analysis.

Microwave-assisted extraction is another extraction technique that, as the name suggests, uses microwave energy to heat the solvents in contact with the sample with the aim of transferring the analytes from the matrix into the solvent. It is suitable for routine analyses and allows for a significant reduction in time and solvent consumption, as well as enabling a high sample extraction throughput simultaneously [127].

### 2.2. Separation and Detection of Phthalates in Wine and Olive Oil

The extraction and cleaning procedures are generally the most critical and challenging aspects in the analysis of phthalates in foods, and both will influence the choice of analytical technique. The physicochemical characteristics of the target analytes and the required sensitivity also determine the suitable instrumental technique for separation, detection, and quantification.

However, establishing separation and detection techniques for phthalates in real samples is a challenge due to matrix interferences. Several traditional analysis techniques are used for the analysis of phthalates in olive oil and wine, but chromatography-based techniques are the most often employed: High-Performance Liquid Chromatography (HPLC) and Gas Chromatography (GC), or more advanced approaches like GC/MS, GC/MS/MS, GC×GC/MS, UHPLC/MS/MS, and LC/MS, due to their sensitivity, separation, and identification capabilities. Other techniques, such as enzyme-linked immunosorbent assay (ELISA), Raman spectroscopy, IPCR, and FI-CL, have also been employed.

#### 2.2.1. Gas Chromatography

It is undeniable that the most widely used analytical technique for the analysis of phthalates in olive oil and wine is gas chromatography, primarily coupled with mass spectrometry (GC/MS), given the thermal stability and volatile nature of phthalates.

GC is a separation and analysis technique for mixtures of volatile substances, equipped with an injector where the sample is vaporized, followed by a capillary column where the sample is carried by a mobile phase and separated according to volatility and/or polarity (depending on the nature of the stationary phase), and a detector [128].

Considering the trace presence of these contaminants in food matrices, the splitless injection mode is typically selected to achieve high sensitivity levels. However, their high boiling points make the analysis challenging, as they can decompose during injection. To prevent this and improve vaporization efficiency, the injector temperature is set to be similar to the boiling points of the PAEs [45,129].

One solution to avoid these two problems is the use of a programmed temperature vaporizer (PTV) injector, which mitigates discrimination in the injector, analyte decomposition, and increases the amount of sample injected into the column, thus achieving better sensitivity and lower limits of detection (LODs) [130]. Russo et al. used the PTV method for wine sample injection to determine six phthalates, obtaining LODs between 0.2–14 ng/mL [64].

Moreover, not only for aqueous samples, another significant advantage of using the PTV method is related to oily matrices, as this method can mitigate the problem of poor extract clean up, such as in olive oil, thus avoiding system contamination [101]. With PTV and the application of a backflush system, the sample pre-treatment can be reduced to a dilution, minimizing the risk of cross-contamination [74].

As seen in Table 3, the capillary columns used in GC are composed of fused silica, known for their high separation efficiency. The choice of column depends on the nature of the target analytes. Due to the nonpolar nature of phthalates, nonpolar fused silica capillary columns, such as those with 5% phenyl-95% dimethylpolysiloxane phases, are commonly used for separation.

The value of GC for the analysis of phthalates in complex matrices such as olive oil and wine is closely related to the availability of increasingly selective and sensitive mass detectors.

According to most of the research reported in Table 3, the analysis of phthalates is performed using GC coupled with Flame Ionization Detector (FID) or Mass Spectrometry (MS).

However, GC/MS is the most commonly reported technique due to its high sensitivity and specificity, allowing the detection of these contaminants at very low levels. For example, Cinelli et al. quantified six phthalates using GC-FID, which is a cost-effective, readily available, and easy-to-operate method. However, they used GC/MS with an ion trap mass analyzer for confirmation of peak identification [55].

Nowadays, there are various mass analyzers available, such as Single Quadrupole (Q), which works with Selected Ion Monitoring (SIM), and Triple Quadrupole (QqQ), which works with Multiple Reaction Monitoring (MRM). Both modes reduce the need for chromatographic separation and, to some extent, increase sensitivity. The latter mode has become more common due to its improved sensitivity, although some authors have preferred SIM mode, as both modes showed similar sensitivity [131].

As observed in Table 2, GC/MS has been used by several authors to determine phthalates in olive oil and wine, achieving LOQs in the range of µg/L or less.

From an analytical perspective, phthalates are molecules that present several challenges, from the care required throughout the experimental procedure to prevent cross-contamination to their identification and quantification. Since all phthalates are derived from phthalic acid, there is low specificity among them due to all mass spectra being dominated by the base peak *m*/*z* = 149, making it very difficult to separate them when they elute at the same retention time in the chromatogram.

Barp et al. determined 10 phthalates in olive oil, including DINP and DIDP, two phthalates regulated by EFSA. In this study, it was shown that DINP and DIDP partially coelute due to being composed of several structural isomers. Therefore, it was necessary to quantify them together as a sum, as suggested by regulations [80].

Coelutions and poor resolutions are commonly reported for these two phthalates, and when quantified, they often have higher limits of quantification than other phthalates that elute as a single peak. Additionally, in the same retention time as these two plasticizers, geranylgeraniol, a compound from the oily matrix, also elutes [80].

When it comes to chromatographic interferences in olive oil, squalene, present in quantities of 2000 to 4000 mg/kg, is considered a potential problem. To separate DEHP and DHP from the overloaded squalene, Fiselier et al. employed an analytical approach of thermal desorption of a diluted oil sample in the GC injector. In summary, the diluted oil is injected directly in splitless mode under desorption conditions for phthalates, with the oil layer retained on the liner wall. Subsequentially, a pre-column with a thin layer of a special material was used before the main analysis in chromatography. At the end of each analysis, a technique called “backflushing” was employed to push out and remove heavy compounds that tend to stay in the precolumn. This was performed through a specific exit designed for this purpose [132].

With that said, it is safe to say that the analysis of phthalates is a significant challenge, and therefore, more powerful separation techniques have been suggested.

In the last decade, one of the major trends in gas chromatography has been the combination of independent techniques to enhance the resolving power. The development and application of multidimensional gas chromatography (MDGC), particularly two-dimensional gas chromatography (GC×GC) coupled with mass spectrometry, has been reported.

GC×GC consists of two orthogonal mechanisms based on the use of two capillary columns coated with different phases, which separate sample constituents in a single analysis. The two columns are connected in series by a modulator interface, allowing small portions (a few seconds) of the first dimension (1D) to elute and be cryo-focused onto the second column (2D). Compounds coeluting in 1D undergo further separation in 2D, often resolving coelutions [133].

Therefore, there is great potential for separation, which makes GC×GC/MS have numerous advantages over conventional one-dimensional GC, such as higher peak capacity, improved resolution of thousands of peaks, acquisition of unique structured chromatograms and mass spectra with high sensitivity, and the ability to reduce matrix-related interferences [134]. Thus, it allows the deconvolution of spectra from coeluted peaks. This technique has already been successfully used for various complex matrices, such as food and environmental samples [135,136].

Arena et al. developed a direct method for the analysis of four phthalates in vegetable oils without any sample preparation, using cryogenic modulation GC×GC coupled to a triple quadrupole. With this analytical technique, it was possible to quantify the four phthalates, including the problematic DINP and DIDP pair, where cases of coelution were spectrally resolved [75].

However, this latter technique is considerably more expensive in terms of both purchase and operation, as well as being more complex.

In summary, without a doubt, GC/MS is a superior technique that measures the mass-to-charge ratio of ions produced in the sample. It is the interface of the technique, typically electron ionization (EI)—a strong ionization method—that is responsible for the extensive fragmentation of molecules like phthalates. As a result, highly reproducible mass spectra of each molecule are obtained using the standard ionization energy of 70 eV, regardless of the chosen chromatographic conditions. Therefore, it is possible to identify compounds by comparing them with thousands of spectra available in standard database libraries.

#### 2.2.2. Liquid Chromatography

As mentioned earlier, GC serves as the primary separation method for the analysis of phthalate esters (PAEs). Nonetheless, liquid chromatography (LC) emerges as a dependable substitute for GC, particularly when assessing isomeric blends like DINP and DIDP, offering enhanced selectivity [137].

In this regard, HPLC has been the most commonly used modality, although ultra-high-performance liquid chromatography (UHPLC) has also been applied (Table 3). In LC, the PAEs are injected and dissolved in a mobile phase, passing through a stationary phase where they are separated and subsequently detected [138].

To achieve adequate chromatographic separation and improve the sensitivity of the method, it is crucial to choose appropriate mobile and stationary phases [138]. Gradient elution is generally applied due to differences in the physicochemical properties of PAEs, and mixtures of ACN/water and MeOH/water have been the most common mobile phases for proper separation of these analytes. Regarding the stationary phases, the approach generally involves using a reverse-phase system, and C18 has by far been the most applied stationary phase for the separation of PAEs in wines and olive oils due to the non-polar nature of these compounds. Shorter-chain columns, like C8, have also been selected.

As for detectors, diode-array (DAD) has sometimes been used for the analysis of phthalates in olive oil and wine, but its identification capability is unsatisfactory when compared to mass spectrometric detectors. With the significant advancement of the latter, MS/MS has become the most robust approach for the analysis of these analytes, as they are present in very complex matrices with various interferents.

Electrospray ionization (ESI) has been the most commonly reported, and MS analyzers, such as single quadrupole, triple quadrupole, high-resolution time-of-flight, and q-Orbitrap have also been applied, achieving LODs at the μg/L or μg/kg level in all studies.

Hayasaka et al. successfully applied a simple, practical, and robust HPLC/MS/MS method without sample extraction or enrichment for the analysis of nine phthalates in wine, which prevents or significantly reduces the effect of contamination by leaching from laboratory materials. The group used a retention column placed upstream of the injection valve to retain contaminants in the system, avoiding coelutions. Additionally, they used various internal standards, in this case, deuterium-labeled phthalate esters, to avoid quantification issues (matrix effects), obtaining LODs between 1.6–26.6 µg/L [53].

Vavrous et al. applied the same chromatographic technique as the previous group for the determination of eight phthalates in edible oils, including olive oil. The group performed LLE followed by SPE to remove the major matrix components, attempting to minimize sample handling to avoid cross-contamination. They also equipped their analytical system with a contamination trap, as the previous group did, achieving similar LODs of 5.5–110 µg/kg [90].

These studies had the advantage over GC of achieving lower LODs for phthalate isomeric mixtures, such as DINP and DIDP. As mentioned earlier, these compounds are one of the major challenges when it comes to phthalate monitoring and were confirmed to be as common as DEHP, the most abundant phthalate in real matrices. Therefore, these mixtures become promising targets for future efforts in the application of this chromatographic technique.

Frequently, one often handles LC and GC as competing techniques; however, if one considers exploring and combining both techniques (LC–GC), one can obtain the most of both. Thus, one can employ LC to isolate compounds, given its high sampling capacity, and subsequently direct the eluate to GC analysis, where compounds are separated with higher resolution and sensibility, which can be improved by means of mass spectrometry (MS) [139,140].

This technique is typically applied to complex matrices, and the use of LC helps to eliminate or reduce the time-consuming sample preparation step, minimizing the need for manipulation and thereby reducing the risk of compound loss and cross-contamination, which is crucial in the analysis of phthalates.

LC–GC is frequently employed for the analysis of various analytes in both wines and olive oils [141,142]. However, concerning the analysis of phthalates using LC–GC, one can only find applications to water samples [143,144]. To date, to the best of our knowledge, no studies have been found that apply it to the analysis of phthalates in olive oil and wine. Therefore, it would be meaningful to explore this possibility.

#### 2.2.3. Other Analytical Techniques

Although the chromatographic methods mentioned above are suitable and accurate for the analysis of phthalates in olive oils and wines, they typically require laborious, time-consuming, and costly procedures.

Therefore, there is a continuous search for alternative methods that are simple, fast, and cost-effective, preferably allowing for improved LODs and LOQs.

Biochemical tests that apply enzymes or antibodies as identification components have been receiving increasing attention due to their high specificity and sensitivity. Examples include the Enzyme-Linked Immunosorbent Assay (ELISA) and Immuno Polymerase Chain Reaction (iPCR).

The chemiluminescence-based ELISA (icELISA) method was applied for the detection of DBP in wine, achieving an LOD of 64.5 ng/mL and recoveries between 83.5–101.7%. When compared to GC/MS, it obtained a correlation of 0.928 in detecting a real sample [50]. On the other hand, the real-time immuno-PCR (rt–IPCR) method was applied for the detection of DMP, and the results were consistent with those obtained by GC/MS [52].

However, despite their precision and reliability, these methods are highly specific and limit the number of phthalates that can be analyzed at once since they are designed for a single specific target molecule.

Table 3 also shows other methods, such as flow-injection chemiluminescence (FI-CL), Supercritical Fluid Chromatography with Ultraviolet (SFC-UV) detection, and Surface-Enhanced Raman Spectroscopy (SERS) technology combined with chemometrics have also been used to determine phthalates in olive oil and wine. Colorimetric methods are generally more basic and user-friendly, and when combined with nanomaterials, they can provide highly sensitive results due to their selectivity [145].

### 2.3. The Major Challenge in the Laboratory Analysis of Phthalates

Due to the widespread use of products containing phthalates, these contaminants have become omnipresent everywhere, including analytical laboratories. The low cost of plastic materials has led to their use in various laboratory applications, making them a considerable problem during sampling, sample preparation, extraction, and instrumental analysis. Besides causing contamination issues with blanks, they increase the risk of cross-contamination, leading to background signals that complicate the analysis of real samples.

Nonetheless, it is not solely plastic materials, such as pipette tips or storage containers, that have the potential to contaminate the sample. Various other laboratory components, including solvents, chemical sorbents, water, glassware, and even ambient air and dust within the laboratory, can harbor phthalates [146,147,148,149]. DBP and DEHP were the most frequently found contaminants. For example, Fankhauser-Noti et al. detected laboratory air at concentrations of 3 and 2.4 µg/m^3^. It was even estimated that a 1.5 mL autosampler vial contains 10 ng of DBP and 4 ng of DEHP [146]. Phthalates were also found in small amounts in Milli-Q water [148]. In fact, even in high-purity organic solvents used for the extraction and analysis of PAEs in foods, phthalates were found at levels of up to mg/L [147]. The authors illustrated that the primary concern in phthalate analysis does not lie in the analysis process itself but rather in the susceptibility to contamination at various stages of the analytical procedure. Such contamination can potentially result in false positives or overestimation of results.

To avoid such contaminations, different strategies have been adopted by analysts. It is recommended that the analysis of PAEs is conducted in a separate area of the laboratory, preferably one with air filters, and that plastic materials in all procedures be replaced with glass, Teflon, PTFE, aluminum, or stainless steel [150,151]. However, it is known that this is not sufficient, and other measures are still recommended. Starting with glass materials, which should be washed with solvents and heated to 400 °C for several hours [146]. Glass materials that cannot be cleaned by heating should be washed with pure solvents taken from containers to which aluminum oxide (oxidizing agents) has been added [152]. The materials required for analysis, such as sample vials, should, whenever possible, be stored in desiccators containing aluminum oxide and/or covered with aluminum foil [153]. Alternatively, they can be stored in suitable glass or PTFE containers to prevent the adsorption of PAEs from the air.

Checking for the absence of PAEs in gloves and pipette tips is crucial. Caps of vials, extraction cartridges, syringes, filters, and septa should also be checked for the presence of PAEs before the start of the analytical procedure [146].

It is also recommended to avoid personal care products by the analysts. Creams, perfumes, and lotions may contain significant amounts of PAEs.

Finally, to track all possible contamination routes, it is essential to perform analytical blanks for each stage of the analytical procedure simultaneously with the set of samples analyzed, preferably in triplicate [154]. Blanks are expected to be free of PAEs to ensure that no contamination occurs during the procedure. Additionally, if high contamination levels are expected, increasing the number of blanks is recommended [150].

In addition to blanks, another recommended measure in the quantification of phthalates is the use of internal or external standards [154]. These measures are advisable because of the multiple stages involved between sampling and the ultimate analysis. These stages encompass extraction, purification, pre-concentration, transfer, and storage, where target analytes can be lost, such as more volatile phthalates. Thus, the use of internal standards allows for the correction of both the potential loss of target analytes throughout the procedure, as well as variations in the injected volume, detector response, and matrix effects, ensuring greater precision in the analysis.

Therefore, a well-selected internal standard, for example, isotopic ISs, along with blank analysis, is crucial to ensure accuracy and precision in quantifications.

## 3. Conclusions and Future Perspectives

Phthalates, known to migrate from polymers into food, require strict measures and regulations. First and foremost, the careful selection of materials for both industrial machinery and packaging during food production is essential to reduce the potential risk of migration. Furthermore, specific limits for phthalates in food should be related to already regulated migration limits in food packaging. Currently, only five phthalates (DBP, BBP, DEHP, DINP, and DIDP) are regulated, even though studies indicate the presence of other phthalates in food, some exceeding regulated limits. With the 2023 update to Regulation (EU) 10/2011, specific migration limits were lowered, raising concerns regarding these plasticizers.

The chemical properties of phthalates pose analytical challenges in sample preparation, identification, and quantification. Additionally, detection limits are very low, and cross-contamination is a concerning factor.

Advances in sample preparation techniques, such as SPME and DLLME, align with the principles of “Green Analytical Chemistry,” offering simpler, faster methods with reduced solvent usage. However, no technique is considered suitable for a complete analysis of phthalates in complex matrices like olive oil and wine.

The analysis of phthalates in olive oil and wine primarily relies on conventional methods, such as GC and LC coupled with various detectors, with mass spectrometry being the primary choice due to its exceptional capabilities for identification and quantification. Emerging techniques, like GC×GC/MS and LC-GC/MS, show significant potential to enhance phthalate analysis.

It is important to note that, as certain phthalates face restrictions and increased scrutiny from the scientific community, alternative compounds are emerging, such as terephthalates, trimellitates, adipates, and sebacates. However, the migration of these compounds into food and their impact on human health remains uncertain. It is imperative to subject these alternatives to epidemiological studies to assess their effects on health and explore potential analytical methods for future controls.

## Figures and Tables

**Table 1:** Physical–chemical properties and applications of various PAEs. Data from PubChem.

Name	Molecule	CAS	Molecular Structure	Molecular Weight (g/mol)	Density (g/cm^3^)	Melting Point (°C)	Boiling Point (°C)	Solubility (mg/L in Water)	Applications
Bis(2-ethylhexyl) phthalate DEHP	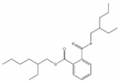	117-81-7	C_24_H_38_O_4_	390.6	0.981 (25 °C)	−50	384	0.27 (25 °C)	Used as a plasticizer; also used in pesticides (an inert ingredient), dielectric fluids, erasable inks, and vacuum pump oils;
Dimethyl phthalate DMP	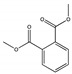	131-11-3	C_10_H_10_O_4_	194.2	1.194 (20 °C)	5.5	284	4 (25 °C)	Used as a plasticizer in solid rocket propellants, lacquers, plastics, safety glasses, rubber coating agents, molding powders, insect repellents, and pesticides.
Diisodecyl phthalate DIDP	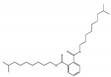	26761-40-0	C_26_H_46_O_4_	446.7	0.966 (20 °C)	−58	53	0.28 (25 °C)	Used as a plasticizer for polyvinyl chloride in calendered film, coated fabrics, building wire jackets, wire, and cable extrusion.
Benzyl butyl phthalate BBP	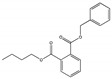	85-68-7	C_19_H_20_O_4_	312.4	1.119 (25 °C)	−35	370	2.69 (25 °C)	Used as an organic intermediate and a plasticizer for PVC-based flooring products, polyvinyl acetate emulsion adhesives, polyvinyl and cellulose resins, vinyl foams, and other plastics.
Dibutyl phthalate DBP	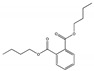	84-74-2	C_16_H_22_O_4_	278.3	1.049 (20 °C)	−35	340	11,2 (25 °C)	Used as a plasticizer to help make plastics soft and flexible; also used in shower curtains, raincoats, food wraps, bowls, car interiors, vinyl fabrics, and floor tiles.
Dioctyl phthalate DOP	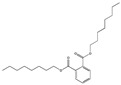	117-84-0	C_24_H_38_O_4_	390.4	0.978 (20 °C)	−25	220	0.022 (25 °C)	Used as a plasticizer in carpet backing, packaging films, medical tubing, blood storage bags, floor tile, wire, cables, adhesives, cosmetics, and pesticides.
Diisononyl phthalate DINP	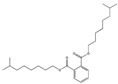	28553-12-0	C_26_H_42_O_4_	418.6	0.972 (20 °C)	−48	78	0.2 (20 °C)	Used to impart softness and flexibility to PVC products. Used in perfumes and cosmetics, vinyl swimming pools, plasticized vinyl seats, and clothing.
Diisobutyl phthalate DIBP	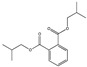	84-69-5	C_16_H_22_O_4_	278.3	1.05 (15 °C)	−64	296	6.2 (25 °C)	Used as a plasticizer; used in paints, lacquers, and varnishes, in the paper and pulp industry, and to make boards, chemicals, polymers, adhesives, softeners, and viscosity adjusters.
Diethyl Phthalate DEP	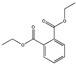	84-66-2	C_12_H_14_O_4_	222.2	1.12 (20 °C)	−41	295	1.08 (25 °C)	Used as a plasticizer, insect repellent, and solvent; as a solvent in cellulose acetate, fragrances, and cosmetics;
Dipropyl phthalate DPrP	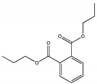	131-16-8	C_14_H_18_O_4_	250.3	1.07 (25 °C)	−31	317.5	108.1 (20 °C)	Used to make plasticizers and polymer additives. It is also used in chemical reagents and organic intermediates.
Diphenyl phthalate DPhP	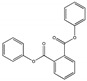	84-62-8	C_20_H_14_O_4_	318.3	1.28 (TNS)	75	402.5	0.082 (24 °C)	Used as a plasticizer in nitrocellulose lacquers.
Bis(2-butoxyethyl) phthalate DBEP	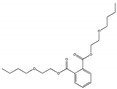	117-83-9	C_20_H_30_O_6_	366.4	1.06 (20 °C)	−55	270	1.675 (25 °C)	Used as a plasticizer for resins, and as a softener and processing aid for chloroprene rubber, nitrile-butadiene rubber, and styrene-butadiene rubber.
Diisopentyl phthalate DIPP	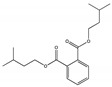	605-50-5	C_14_H_18_O_6_	306.4	1.02 (TNS)	<−25 °C	339	1.1 (20 °C)	Used as plasticizer of cellulose resin, polymethyl methacrylate, polystyrene, and chlorinated rubber.
Bis(4-methyl-2-pentyl) phthalate BMPP	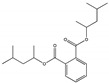	84-63-9	C_20_H_30_O_4_	334.4	0.995 (TNS)		341	<0.1%	Used as a plasticizer and found in cosmetics and baby skin care products.
Diallyl phthalate DAP	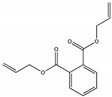	131-17-9	C_14_H_14_O_4_	246.3	1.12 (20 °C)	−70	290	182 (25 °C)	Used to make insulators, potentiometers, and circuit boards in communication, computer, and aerospace systems, and a monomer in thermosetting plastics, a diluent in polyester spray systems, a dye carrier, and an impregnant for jewelry.
Dihexyl phthalate DHXP	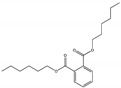	84-75-3	C_20_H_30_O_4_	334.4	1.01 (20 °C)	−59	350	0.05 (25 °C)	Used as a plasticizer; used to make plastisols for automobile parts and dip-molded products.
Diheptyl phthalate DHP	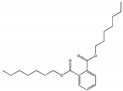	3648-21-3	C_24_H_34_O_4_	362.5	1 (20 °C)	<−40	360	0.0018 (25 °C)	Used as a plasticizer for vinyl resins.
Dipentyl phthalate DPP	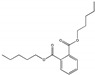	131-18-0	C_18_H_26_O_4_	306.4	1.12 (20 °C)	<−55	342	0.8 (25 °C)	Used as plasticizers to soften polyvinyl chloride in shower curtains, vinyl upholstery, adhesives, floor tiles, food containers and wrappers, cleaning materials, and cosmetics.
Dicyclohexyl phthalate DCHP	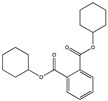	84-61-7	C_20_H_26_O_4_	330.4	1.383 (20 °C)	66	224	4.0 (24 °C)	Used as a plasticizer for nitrocellulose, ethyl cellulose, chlorinated rubber, polyvinyl acetate, polyvinyl chloride, and other polymers; And as a heat sealer for cellulose, in paper finishes, and to make printers ink water-resistant;
Bis(2-ethoxyethyl) phthalate DEEP	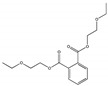	605-54-9	C_16_H_22_O_6_	310.3	1.121 (20℃)	34	345	1946 (TNS)	Used as a plasticizer, an apoptosis inhibitor, and an androstane receptor agonist.
Dinonyl phthalate DNP	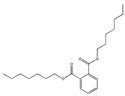	84-76-4	C_26_H_42_O_4_	418.6	0.972 (20 °C)	−33.15	413	1.73 × 10^−5^ (25 °C)	Used in plastisols and coating pastes, as a low-volatility plasticizer for vinyl resins, as a stationary liquid phase in chromatography, and to make vinyl mixes resistant to heat and detergents;
Bis(2-methoxyethyl) phthalate DMEP	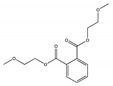	117-82-8	C_14_H_18_O_6_	282.3	1.1596 (15 °C)	−45	340	8500 (25 °C)	Used in plastisols and coating pastes, as a plasticizer for vinyl resins, as a stationary liquid phase in chromatography, and to make vinyl mixes resistant to heat and detergents.
Bis(2-propylheptyl) phthalate DPHP	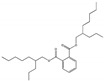	53306-54-0	C_28_H_46_O_4_	446.7	0.964 (TNS)	−48	254	2 × 10^−7^ (25 °C)	Used as an adhesion/cohesion promoter, adhesives and sealant chemicals, intermediate, paint additives, and coating additives.

**Table 2:** Comparison between regulation 10/2011 and its amendment 2023/1442.

Substance	Regulation (EU) 2023/1442 Amending Annex I to Regulation (EU) 10/2011 *	Annex I to Regulation (EU) 10/2011	Only to Be Used as:
DBP	SML: 0.12 mg/kg Total SML group restriction no.32: 60 mg/kg Total SML group restriction no.36: 0.6 mg/kg	SML: 0.3 mg/kg Total SML group restriction no.32: 60 mg/kg	(a) Plasticizer in repeated use materials and articles contacting non-fatty foods; (b) Technical support agent in polyolefins in concentrations up to 0.05% (*w*/*w*) in the final product.
BBP	SML: 6.0 mg/kg Total SML group restriction no.32: 60 mg/kg Total SML group restriction no.36: 0.6 mg/kg	SML: 30 mg/kg Total SML group restriction no.32: 60 mg/kg	(a) Plasticizer in repeated use materials and articles; (b) Plasticizer in single-use materials and articles contacting non-fatty foods except for infant formula and follow-on formula; (c) Technical support agent in concentrations up to 0.1% (*w*/*w*) in the final product.
DEHP	SML: 0.6 mg/kg Total SML group restriction no.32: 60 mg/kg Total SML group restriction no.36: 0.6 mg/kg	SML: 1.5 mg/kg Total SML group restriction no.32: 60 mg/kg	(a) Plasticizer in repeated use materials and articles contacting non-fatty foods; (b) Technical support agent in concentrations up to 0.1% (*w*/*w*) in the final product.
DINP and DIDP	Total SML group restriction no.26: 1.8 mg/kg (sum of DINP and DIDP) Total SML group restriction no.32: 60 mg/kg Not to be used in combination with FCM substances DBP, BBP, DEHP, and DIBP.	Total SML: 9 mg/kg (sum of DINP and DIDP) Total SML group restriction no.32: 60 mg/kg	(a) Plasticizer in repeated use materials and articles; (b) Plasticizer in single-use materials and articles contacting non-fatty foods except for infant formula and follow-on formula; (c) technical support agent in concentrations up to 0.1% (*w*/*w*) in the final product.

* Group restriction no. 26 corresponds to the sum of DINP e DIDP; Group restriction no. 36 corresponds to the sum of DBP, DIBP, BBP, and DEHP expressed as DEHP equivalents using the following equation: DBP*5 + DIBP*4 + BBP*0,1 + DEHP*1; Group restriction no. 32 corresponds to the sum of DBP BBP DEHP DIBP and some plasticizing substances like adipates, sebacates, and terephthalates, among others. DIBP is not listed as an authorized substance; however, it may occur alongside other phthalates as a result of its use as a polymerization aid, and therefore, it is included in group restrictions.

## Data Availability

Not applicable.
